# Integrated UPLC–MS/MS and UPLC–Q–TOF–MS/MS analysis to reveal pharmacokinetics, tissue distribution, metabolism, and excretion of sipeimine in rats

**DOI:** 10.3389/fphar.2025.1595731

**Published:** 2025-05-30

**Authors:** Hui Zong, Chongyang Wang, Dongdong He, Liting Liu, Juanjuan Wan, Guodong Wang, Dingtao Li, Jun Ran, Meiling Zhang, Hui Tang, Liping Wang

**Affiliations:** ^1^ Key Laboratory of Xinjiang Phytomedicine Resource and Utilization, Ministry of Education, School of Pharmacy, Shihezi University, Shihezi, Xinjiang, China; ^2^ Chemistry Room, Eighth Division Shihezi City Inspection and Testing Center, Shihezi, Xinjiang, China

**Keywords:** sipeimine, pharmacokinetics, tissue distribution, metabolism, excretion

## Abstract

**Background and Objective:**

*Fritillaria* Bulbus is a traditional Chinese medicine used to treat respiratory diseases such as cough, expectoration, and asthma for more than 2000 years. Sipeimine, a major isosteroidal alkaloid isolated from *Fritillaria* Bulbus, has attracted considerable attention from the research community owing to its antitussive, anti-inflammatory, and lung-protective activities. However, there exist few reports regarding the *in vivo* disposition of sipeimine. This study aims to investigate the disposition of sipeimine in rats.

**Methods:**

A rapid, sensitive, and selective UPLC–MS/MS approach was developed to the quantification of sipeimine in various biological samples and successfully applied to the investigation of pharmacokinetic characteristics, tissue distribution, and excretion of sipeimine in rats. A reliable UPLC–Q–TOF–MS/MS combined with a scientific metabolite identification strategy was used to characterize the metabolic transformation of sipeimine in rat plasma and urine.

**Results:**

The established UPLC–MS/MS method was accurate and reliable with a good linearity (r^2^ > 0.99) in the respective concentration range, satisfying the quantitative requirements. Sipeimine exhibited the characteristics of rapid absorption and slow elimination in rats, with an average oral bioavailability of 40%. Furthermore, sipeimine was rapidly distributed in all the organs except brain, and the plasma protein binding ratio of sipeimine was found to be approximately 30%. The metabolism of sipeimine in rats is chiefly accomplished via its hydroxylation, sulfation, and glucose conjugation. Analysis of fecal and urinary samples revealed that sipeimine is predominantly excreted unchanged via renal elimination.

**Conclusion:**

The pharmacokinetics, tissue distribution, metabolism, and excretion of sipeimine were comprehensively characterized and elucidated. These results are expected to prove useful for the interpretation of the pharmacokinetic and pharmacodynamic characteristics of sipeimine and the traditional Chinese medicines containing sipeimine.

## 1 Introduction

*Fritillaria* Bulbus, referred to as “Bei-mu” in Chinese, holds significance not only as a botanical resource but also as a source of food and medicine worldwide (Kumar et al., 2024). There are five sorts of *Fritillaria* Bulbus officially recorded in Chinese Pharmacopeia (2020 edition), including *Fritillaria cirrhosa* D.Don, *Fritillaria thunbergii* Miq., *Fritillaria usuriensis* Maxim., *Fritillaria pallidiflora* Schrenk ex Fisch. & C.A.Mey., and *Fritillaria monantha* Migo. (The plant name has been checked with http://www.theplantlist.org). According to traditional descriptions, *Fritillaria* Bulbus is slightly cold and affects the lungs (to clear heat and moisten dryness, and used for hot-type bronchitis with dry cough) and the heart (to calm heart fire) (Xu et al., 2019). Modern pharmacological researches have demonstrated that the active components from *Fritillaria* Bulbus have revealed potential in alleviating respiratory disorders, including cough, expectoration, asthma, chronic obstructive pulmonary disease, and nonsmall cell lung cancer (Yeum et al., 2007; Wu et al., 2018; Xu et al., 2019; Liu et al., 2023; Li et al., 2024). At present, isosteroidal alkaloids, saponins, terpenoids, glycosides, and many other compounds have been isolated from *Fritillaria* Bulbus, among which isosteroidal alkaloids are traditionally believed to be responsible for its pharmacological activities (Wang et al., 2012; Wang et al., 2016b; Pai et al., 2023; Peng M. et al., 2023; Wang et al., 2024).

**FIGURE 1:**
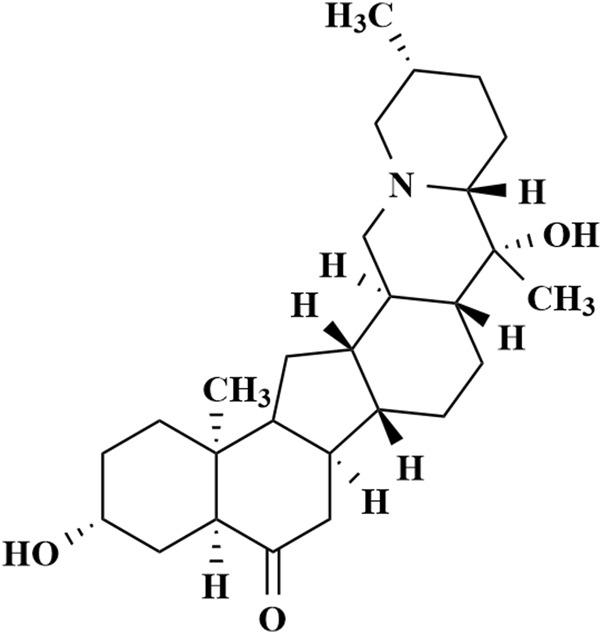
Chemical structure of sipeimine.

Sipeimine, also known as imperialine (Figure 1), is one of the most favored active isosteroidal alkaloids isolated from *Fritillaria* Bulbus and has been shown to exert antitussive, anti-inflammatory, and lung-protective activities (Wu et al., 2015; Wang et al., 2016a; Huang et al., 2023). In LPS-induced inflammatory response, sipeimine reduces inflammatory mediators by inhibiting the PI3K/AKT/NF-κB pathway, thereby suppressing NLRP3 inflammasome activation and pyroptosis, and ultimately alleviating inflammation (Fang et al., 2024). In rats with PM2.5-induced lung toxicity, sipeimine mitigates damage and NLRP3 inflammasome-mediated pyroptosis by regulating the PI3K/AKT signaling pathway (Huang et al., 2023). Moreover, sipeimine exerts anticancer effects against nonsmall cell lung cancer without exhibiting any cytotoxicity and exhibits robust systemic safety (Lin et al., 2020).

The pharmacokinetic profiling of bioactive compounds, including their absorption, distribution, metabolism, and excretion (Sun et al., 2020), is vital for understanding their *in vivo* behavior and mechanisms of action. Currently, most pharmacokinetic studies on *Fritillaria* Bulbus are primarily focused on the extracts (Chen et al., 2013; Wang et al., 2018; Jeong et al., 2024) and other alkaloids (Chen et al., 2015; Wang et al., 2019; Zhang et al., 2021). A few studies have evaluated the behavior of sipeimine in rat plasma using liquid chromatography with evaporative light-scattering detection (Chan et al., 2000) or tandem mass spectrometry (Lin et al., 2013). Sipeimine was determined to be rapidly absorbed from the gastrointestinal tract, with passive membrane diffusion dominating its intestinal absorption (Lin et al., 2013; Lin et al., 2015). Nevertheless, there remains a controversy regarding the oral bioavailability of sipeimine (Chan et al., 2000; Lin et al., 2013). Moreover, the tissue distribution, metabolism, and excretion of sipeimine remain largely unknown.

An ultrahigh performance liquid chromatography–tandem mass spectrometry (UPLC–MS/MS) approach was developed to the quantification of sipeimine in various biological samples and successfully applied to evaluate the pharmacokinetics, tissue distribution, and excretion of sipeimine in rats. Metabolites of sipeimine were identified in rat plasma and urine using UPLC coupled with quadrupole time-of-flight mass spectrometry (UPLC–Q–TOF–MS/MS). These results are expected to prove useful for the interpretation of the pharmacokinetic and pharmacodynamic characteristics of sipeimine and the traditional Chinese medicines containing sipeimine.

## 2 Materials and experimental methods

### 2.1 Chemicals and reagents

Sipeimine (purity ≥ 98%), carbamazepine (Internal standard, IS, purity ≥ 98%), and heparin sodium were obtained from Yuanye Bio-Technology Co., Ltd. (Shanghai, China). Sodium carboxymethyl cellulose (CMC-Na) was obtained from Shanghai Acmec Biochemical Co., Ltd. (Shanghai, China). LC-grade methanol was purchased from Sigma-Aldrich Inc. (St. Louis, United States). LC-grade acetonitrile was purchased from Fisher Scientific International Inc. (Massachusetts, United States). LC-grade formic acid was purchased from Fuchen Chemical Reagent Co., Ltd. (Tianjin, China).

### 2.2 Animals

Male Sprague–Dawley rats weighing 180–220 g were procured from the Henan SkBys Biotechnology Co., Ltd (Anyang, China). All the rats were fed standard lab chow and water *ad libitum* for 7 days and housed under conditions of controlled temperature (21°C ± 2°C), humidity (50% ± 10%), and a 12-h light/dark cycle. Animal procedures adhered strictly to the guidelines set by the Animal Care and Use Committee of the Shihezi University (China), who also approved the study protocol (A2024-025).

### 2.3 Quantification of sipeimine in biological samples from rat using UPLC–MS/MS

UPLC–MS/MS analysis was performed on the ACQUITY UPLC H-Class system (Waters, United States). The chromatographic separation was performed on a Waters ACQUITY UPLC BEH C18 column (100 × 2.1 mm, 1.7 µm) with a mobile phase consisting of 0.1% (v/v) formic acid solution (A) and acetonitrile (B) at a flow rate of 0.3 mL/min. Chromatographic separation was achieved using a gradient elution program, as follows: 0.0–1.0 min, B% 2%–15%; 1.0–2.0 min, B% 15%–25%; 2.0–3.0 min, B% 25%–25%; 3.0–3.1 min, B% 25%–100%; 3.1–4.5 min, B% 100%–100%; 4.5–4.8 min, B% 100%–2%; and 4.8–6.0 min, B% 2%–2%.

Detection by MS was performed on a Waters Xevo TQ-S triple-quadrupole mass spectrometer (Waters, United States) with an electrospray ionization (ESI) source in the positive multiple reaction monitoring (MRM) mode. The parameters were optimized as follows: ion source temperature, 150°C; capillary voltage, 3 kV; desolvation temperature, 450°C; desolvation gas flow, 800 L/h; cone gas flow, 150 L/h; collision gas flow, 0.15 mL/min; precursor–product ion transitions, m/z 430→138 for sipeimine and 237→179 for IS; and declustering potential and collision energy, 100 V and 60 eV for sipeimine and 50 V and 15 eV for IS, respectively. Data acquisition and instrument control was performed using the MassLynx analyst software (Waters, United States).

### 2.4 Preparation of biological samples

In a 1.5 mL centrifuge tube, 10 μL solution of IS was added to 20 μL of biological samples. 200 μL acetonitrile was added to precipitate protein. The resulting solution was subjected to vortex mixing for 3 min and then centrifuged at 16,106 × g for 20 min. The supernatant (200 µL) was transferred to a polypropylene tube and evaporated to dryness by centrifugation at 25°C ± 2°C. The residue was dissolved in 200 µL of 1:1 solution of methanol: acetonitrile, and the resulting solution was again subjected to vortex mixing for 2 min and subsequent centrifugation at 16,106 × g for 20 min. The resulting supernatant was then injected for UPLC–MS/MS analysis.

### 2.5 Method validation

The UPLC–MS/MS method developed herein was fully validated according to the biological sample analysis methodology guide of the United States Food and Drug Administration (FDA), which is described in the Supplementary Material.

### 2.6 Pharmacokinetic studies

All rats were subjected to fasting for 12 h, with free access to water prior to the pharmacokinetics analysis. For intragastric (i.g.) administration, sipeimine was suspended in 0.5% CMC-Na to final concentrations of 0.25, 0.5, and 1 mg/mL. Eighteen rats were randomly divided into three groups (n = 6 per group), and each group received a single oral dose of 2.5, 5, or 10 mg/kg sipeimine. Blood samples (0.5 mL) were collected from the vein of the eye into heparinized tubes at predose and postdose (5, 15, 30, 60, 120, 240, 480, 720, 1,440, and 2,160 min after oral administration) time points. For intravenous (i.v.) administration, sipeimine was dissolved in 0.1% hydrochloric acid solution to a final concentration of 0.25 mg/mL. Six rats received a single intravenous dose of 0.5 mg/kg sipeimine. Blood samples (0.5 mL) were collected at predose and postdose (2, 10, 15, 30, 60, 120, 240, 480, 720, 1,440, and 2,160 min after injection) time points. All the blood samples were centrifuged to obtain the plasma, which was stored at −80°C until further analysis.

To acquire the pharmacokinetic parameters of sipeimine in plasma, the concentration–time data were subjected to noncompartmental analysis using WinNonlin Version 8.0 (Pharsight Corporation, United States). The oral bioavailability (*F*) was calculated by comparing the area under the curve and dose of sipeimine following i.g. and i.v. administration according to the following formula:

$$F\left(\%\right)=\left(AUC_{\text{ig}/}AUC_{\text{iv}}\right)\times \left(Dose_{\text{iv}}/Dose_{\text{ig}}\right)\times 100\%$$

### 2.7 Evaluation of tissue distribution of sipeimine

Twenty-four rats were randomly divided into four groups (n = 6 per group). The rats of the first group were employed for obtaining blank tissue samples. The remaining three groups were sacrificed at 15, 60, and 600 min after the administration of 5 mg/kg sipeimine via the i.g., route. The various tissues, including heart, liver, spleen, lung, kidney, intestines, trachea, and brain, were immediately dissected and washed with physiological saline solution maintained at 4°C. Finally, all the tissues were blotted on filter paper, weighed, and separately homogenized using physiological saline solution to yield the tissue homogenates (1:9, w/v). The concentration of sipeimine in the homogenate samples was determined using UPLC–MS/MS.

### 2.8 *In vitro* evaluation of plasma protein binding ratio

The plasma protein binding ratio of sipeimine was determined using the equilibrium dialysis method. A semipermeable membrane bag (3,500 Da, Beijing Solaibao Technology Co., Ltd., China) was precut to an appropriate size and pretreated according to the instructions. Plasma samples (1 mL) obtained from rats of the blank group were added to the bags, which were then tightly tied at both ends. The bags were then placed in different bottles containing 20 mL of sipeimine solution at three concentrations (50, 250, and 1,250 nM). Five parallel samples were prepared for each concentration. The liquid within and outside the bag was maintained at the same level and care was taken that the bag did not touch the wall of the bottle, the setup was then incubated in a water bath at 37°C for 4 h such that equilibrium was attained. The concentrations of sipeimine in the plasma samples within the bag and the dialysate outside were estimated using UPLC–MS/MS.

### 2.9 Analysis of sipeimine metabolites in rat plasma and urine using UPLC–Q–TOF–MS/MS

The processed plasma and urine samples were injected into a Waters UPLC system equipped with an ACQUITY UPLC BEH C18 column. The mobile phases employed included 0.1% formic acid in water (A) and acetonitrile (B). The following linear gradient program was employed: 0–1 min, B% 5%–5%; 1–3 min, B% 5%–100%; 3–4 min, B% 100%–100%; 4–4.5 min, B% 100%–5%; and 4.5–6 min, B% 5%–5%. The temperature of the column oven was maintained at 40°C, and a flow rate of 0.3 mL/min was employed.

Sipeimine and its metabolites in rat plasma and urine were identified using a SYNAPT XS mass spectrometer (Waters, United States) with an ESI source in positive mode. The working parameters were as follows: source temperature of 120°C, desolvation temperature of 450°C, cone gas flow at 100.0 L/h, and desolvation gas flow at 800.0 L/h. Scanning modes encompassed Full MS/dd-MS_2_. The capillary voltage was 3 kV. The scan range (m/z) was from 50 to 1,000 Da. Collision energy of the second order was set at 50 V.

### 2.10 Evaluation of excretion of sipeimine

Six rats were administered sipeimine (5 mg/kg) via the i.g. route and individually housed in metabolic cages. Urine was collected at 0, 2, 4, 8, 12, 24, and 36 h. Feces were collected at 0, 2, 4, 8, 12, 24, 36, and 48 h. The volumes of the urine samples were measured accurately, followed by their 50-fold dilution. Feces were dried at 37°C, homogenized in 9 volumes (v/w) of 50% methanol/50% water and then centrifuged at 740 × g for 10 min. The concentration of sipeimine in the urine and feces was determined using UPLC–MS/MS.

## 3 Results and discussion

### 3.1 Validation of the UPLC–MS/MS method

#### 3.1.1 Selectivity

The application of the UPLC–MS/MS method was validated for the quantitative analysis of sipeimine in rat plasma and other biological samples. Under the established chromatographic conditions, the analysis of sipeimine and IS using the selected MRM mode was highly selective without any interfering peaks. The typical chromatograms are shown in Supplementary Figures S1, S2.

#### 3.1.2 Linearity and lower limit of quantitation

As shown in Supplementary Table S1, the calibration curves demonstrated good linearity over the concentration ranges typical of sipeimine in rat plasma and other samples, with correlation coefficients (r^2^) ≥ 0.99.

#### 3.1.3 Precision and accuracy

Quality control samples were assessed at three concentrations to evaluate the precision and accuracy of the method. As shown in Supplementary Table S2, The values of relative standard deviation (RSD, for precision) and relative error (RE, for accuracy) were within ±15.0%. These observations demonstrate the reliability and reproducibility of the method developed herein.

#### 3.1.4 Stability

The results of the experiments to evaluate stability are presented in Supplementary Table S3. Sipeimine was found to be stable in biological samples stored at −80°C for 2 weeks and after three cycles of freezing–thawing. In addition, the processed samples were stable at 4°C for 6 h, with RSD and RE values of within ±15%.

#### 3.1.5 Extraction recovery and matrix effect

The extraction recoveries and matrix effects of sipeimine are shown in Supplementary Table S4. The extraction of sipeimine at different concentrations were consistent and a significant matrix effect was not detectable in the biological samples.

### 3.2 Pharmacokinetics analysis

**FIGURE 2:**
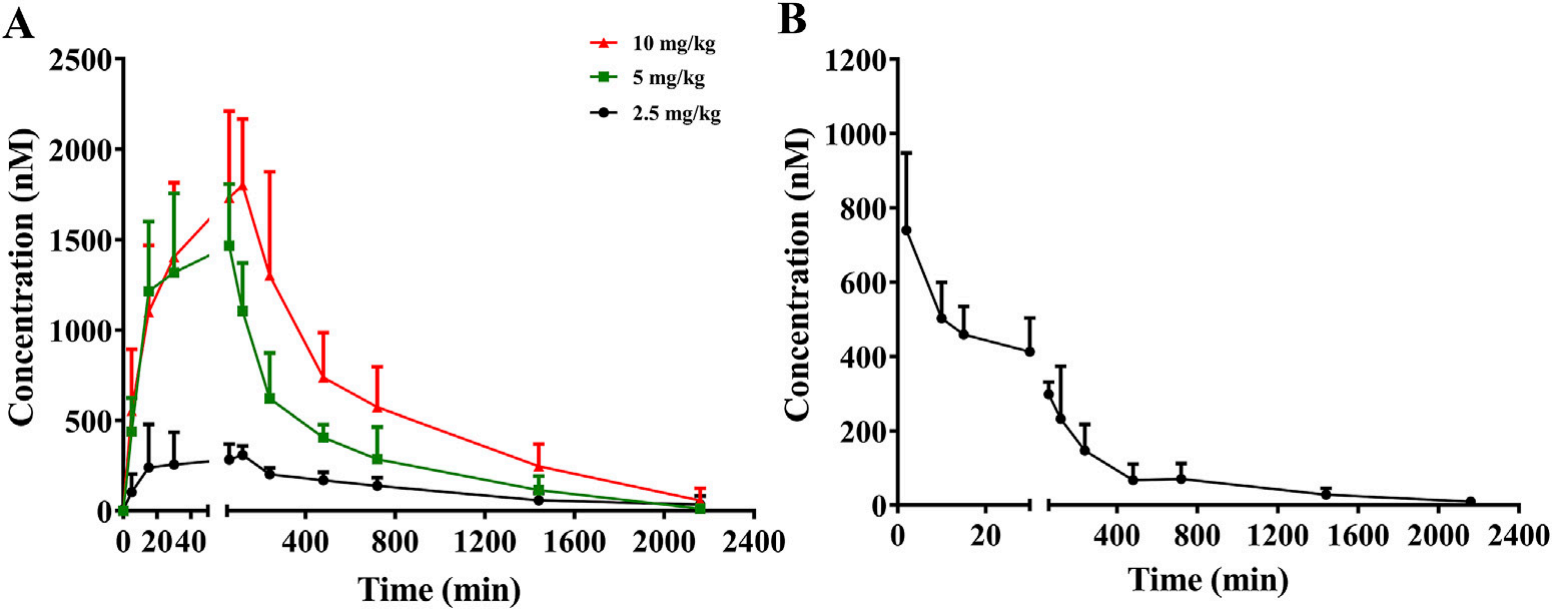
Mean plasma concentration-time curves of sipeimine in rats after intragastric (i.g., A) and intravenous (i.v., B) administration of sipeimine (*n* = 6, Mean ± SD).

The mean plasma concentration–time profiles of sipeimine following its administration via the i.g. and i.v. routes in rats are presented in Figure 2. The estimated pharmacokinetic parameters are summarized in Table 1. Following the oral administration of sipeimine, the time to peak concentration (*t*_max_) of sipeimine ranged from (0.83 ± 0.26) to (1.83 ± 0.41) h, demonstrating its quick absorption in rats. Sipeimine exhibited high levels of systemic exposure and a dose-proportional linear increase in the plasma of rats. Additionally, the values of elimination half-life (*t*_*1/2*_) and mean residence time (*MRT*) indicated that sipeimine persists in the body for a prolonged duration. Our study also observed that the *t*_*1/2*_ of the i.g., (2.5 mg/kg) dose group shows unusually high variability, potentially attributed to amplified inter-individual differences in enzyme activity under unsaturated metabolic conditions, compounded by gastrointestinal absorption fluctuations and detection sensitivity limitations. The values for volume of distribution ([*V*_d_]/*F*) in rats ranged from (9.57 ± 2.34) to (17.31 ± 6.82) L/kg, which indicated that sipeimine was widely distributed in plasma and extracellular fluid. Moreover, a significant local accumulation of sipeimine was not observed, which closely corresponds with the observed pattern of wide tissue distribution.

**TABLE 1:** Pharmacokintic arameters of sipeimine in rats following intragastric (i.g., 2.5, 5, and 10 mg/kg) and intravenous (i.v., 0.5 mg/kg) administration of sipeimine (n = 6, Mean ± SD).

Parameter	i.g. (2.5 mg/kg)	i.g. (5 mg/kg)	i.g. (10 mg/kg)	i.v. (0.5 mg/kg)
*t*_max_(h)	1.29 ± 0.81	0.83 ± 0.26	1.83 ± 0.41	0.03 ± 0.00
*C*_max_ (mg/L)	0.17 ± 0.07	0.65 ± 0.15	0.83 ± 0.15	0.32 ± 0.09
*t*_1/2_(h)	11.83 ± 11.46	5.91 ± 0.76	9.88 ± 2.38	7.99 ± 2.63
*AUC*_0-t_ (h*mg/L)	1.78 ± 0.48	4.60 ± 1.51	8.59 ± 2.60	1.12 ± 0.51
*AUC*_0-_ ∞ (h*mg/L)	2.23 ± 1.22	4.70 ± 1.54	9.70 ± 3.20	1.18 ± 0.53
*MRT*_0-t_(h)	10.64 ± 3.06	7.92 ± 1.76	9.50 ± 1.82	7.98 ± 2.25
*CL/F* (L/h/kg)	1.39 ± 0.69	1.13 ± 0.25	1.17 ± 0.51	0.48 ± 0.17
*V*_d_/*F* (L/kg)	17.31 ± 6.82	9.57 ± 2.34	15.68 ± 4.67	5.34 ± 1.88
*F* (%)	37.80	39.83	41.10	—

For the group that was administered sipeimine via the i.v., route, the maximum plasma drug concentration (*C*_max_) and value of *AUC*_0–t_ were found to be 0.32 ± 0.09 mg/L and 1.12 ± 0.51 h∙mg/L, respectively. The oral bioavailability of sipeimine ranged from 37.8% to 41.1% at the three oral dosages employed, which is in line with the results obtained in a previous study (Lin et al., 2013). The pharmacokinetic characteristics of rapid absorption, slow elimination, and high oral bioavailability reveal that sipeimine can be retained in the body for a prolonged duration and exert therapeutic effects. However, female animals were not included in this study, and it may not be possible to fully predict fluctuations in exposure due to sex differences. Further research on gender differences is worth conducting.

### 3.3 Tissue distribution of sipeimine

**FIGURE 3:**
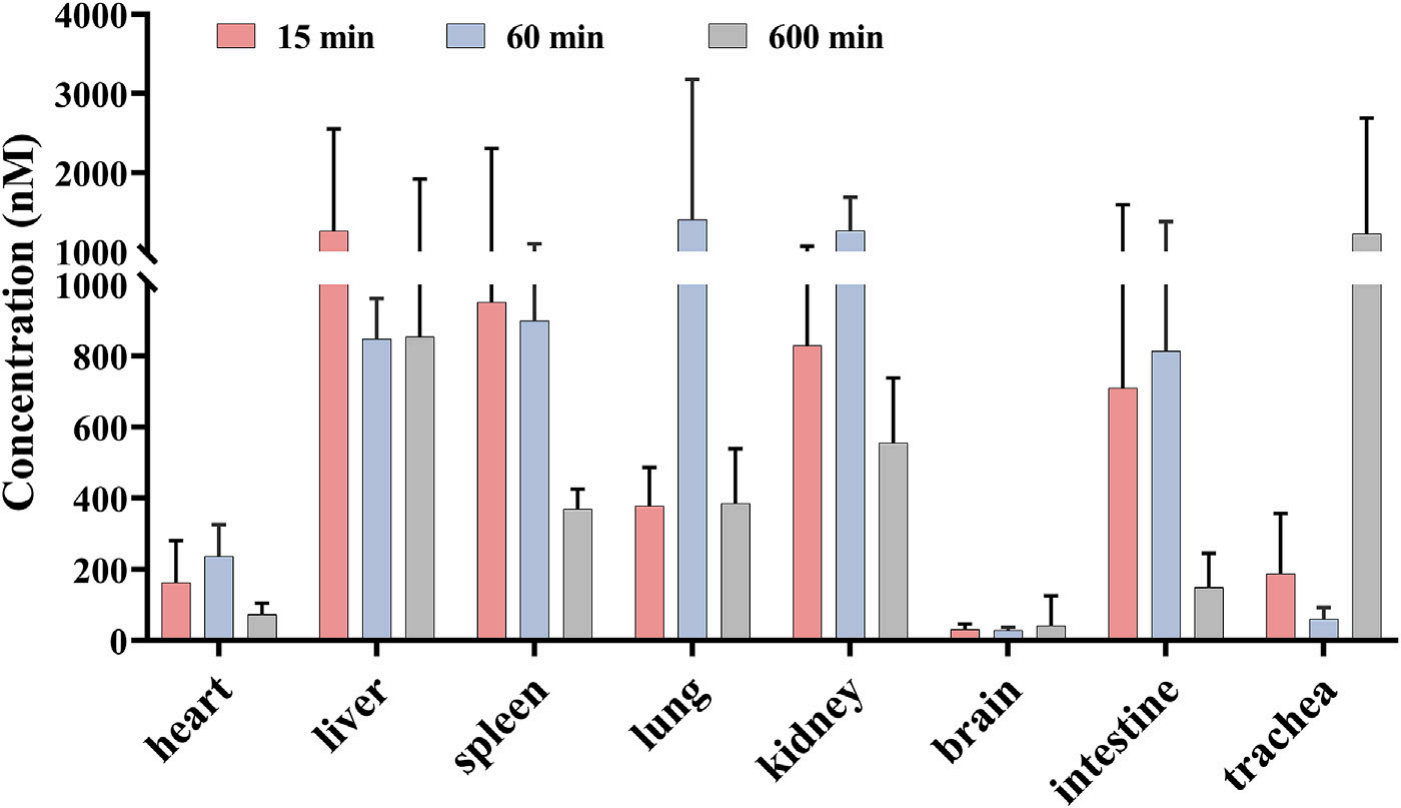
Mean concentration of sipeimine in rat tissues at 15, 60, and 600 min after intragastric administration of 5 mg/kg sipeimine (*n* = 6, Mean ± SD).

Based on the mean plasma concentration–time profiles, the time points 15, 60, and 600 min after the oral administration of sipeimine (5 mg/kg) were selected for the study of its distribution in rat tissues. As shown in Figure 3, the results reveal that sipeimine was rapidly distributed to various tissues following i.g., administration. The concentrations of sipeimine in various tissues at the 15-min time point were determined as follows: liver > spleen > kidney > intestines > lung > trachea > heart > brain. The prominent increase in the concentration of sipeimine in the lung and kidney tissues at the 60-min time point may be associated with its lung-protective effects and renal excretion. At the 600-min time point, the concentration of sipeimine was observed to decrease in most tissues. Interestingly, the concentration of sipeimine in the trachea remained high at the 600-min time point, which is beneficial for effective lung-protective activity. Furthermore, the high concentration and long-term retention of sipeimine in the liver is suggestive of a strong affinity of sipeimine for the liver and low rate of hepatic clearance. In addition, the concentration of sipeimine in the brain was found to be very low, suggesting difficulties in crossing the blood–brain barrier.

### 3.4 *In vitro* evaluation of plasma protein binding ratio

An evaluation of the plasma protein binding ratio of a drug helps explain drug–drug interaction, pharmacodynamics, and pharmacokinetic characteristics, which are closely related to its reversible binding with plasma proteins (Yang et al., 2022). The plasma protein binding ratio affects the pharmacokinetics of unbound drugs, leading to alterations in clinical outcomes (Xiang et al., 2022). As shown in Table 2, the plasma protein binding ratio of sipeimine was found to be approximately 30%, which is considered moderate; this corroborates the pharmacokinetic characteristics of “rapid absorption and slow elimination” exhibited by sipeimine in rats. A saturation of plasma protein binding did not occur within the sipeimine concentration range of 50–1,250 nM, indicating the relatively sufficient abundance of the binding site that interacts with the plasma proteins or the moderate affinity of its molecular structure for these proteins, which is insufficient to support saturation at low concentrations. In addition, the free (unbound) drug remaining in the body following the administration of any drug is chiefly responsible for its efficacy; a moderate extent of plasma protein binding may indicate a certain concentration of free sipeimine in the body, such that a decrease in free drug concentration due to tissue distribution results in the reversible supplementation of the bound drug. This process inevitably delays the elimination of the drug from the body and prolongs the duration of drug activity, which is an important aspect in the evaluation of efficacy and safety of the active ingredient.

**TABLE 2:** The protein binding rates of sipeimine in rat plasma *in vitro* (Mean ± SD, n = 5).

Concentration/nM	Protein binding rates (%)	Average (%)
50	30.32 ± 7.22	29.91 ± 5.90
250	28.46 ± 3.65	
1,250	30.95 ± 6.84	

### 3.5 Identification of sipeimine metabolites

The metabolism of the active ingredient of any drug is of great significance (Peng W. et al., 2023). This study identified the metabolites of sipeimine in rat plasma and urine using UPLC–Q–TOF–MS/MS in the positive-ion mode. A comparison of the blank sample with the samples obtained after drug administration allowed the identification of sipeimine and its three metabolites, which has been explained subsequently. The most abundant of these metabolites in both rat plasma and urine was M1. Sipeimine is readily conjugated with sulfuric acid and glucose owing to the presence of the hydroxy group in the molecule, yielding the sulfated (M2) and glucose (M3) conjugates, respectively.

**FIGURE 4:**
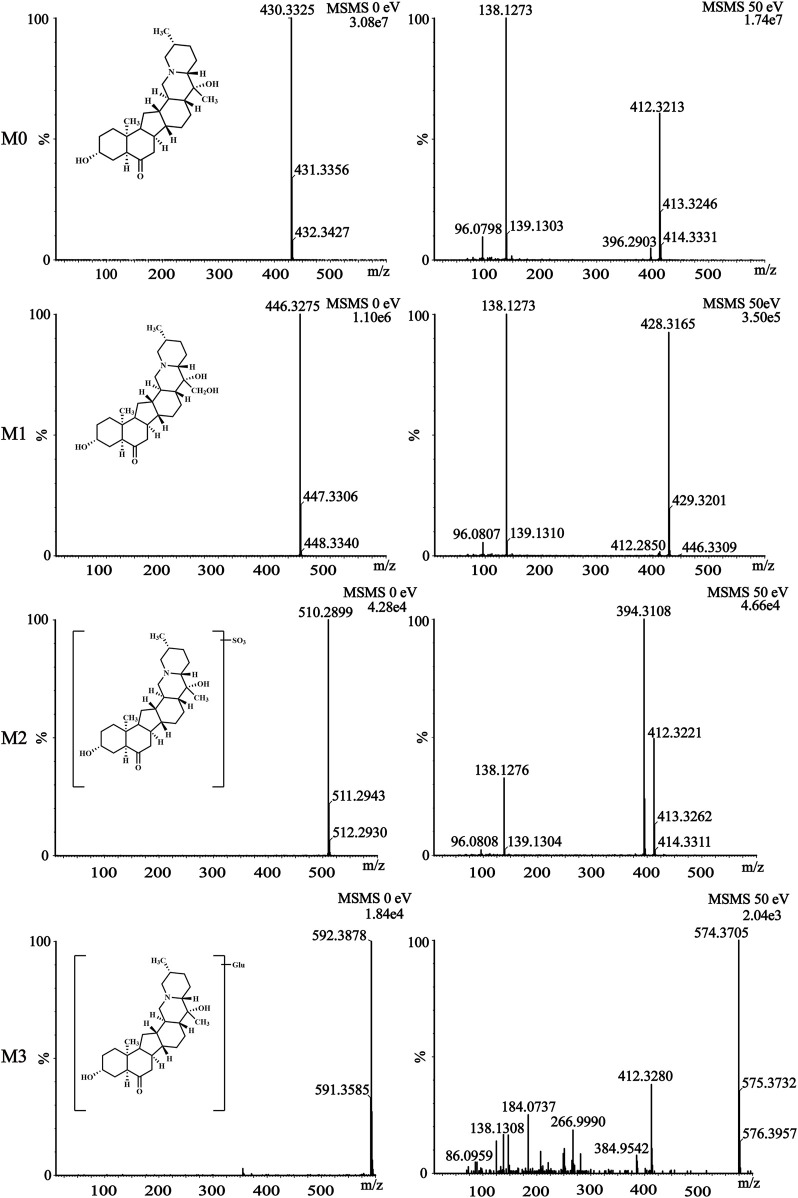
The mass spectra fragmentation of sipeimine (M0) and its metabolites (M1, M2, and M3) in rat plasma and urine after intragastric administration of 5 mg/kg sipeimine.

The parent compound sipeimine (M0) was eluted at 2.213 min and was detected as [M + H]^+^ at an m/z of 430.3325 (C_27_H_43_NO_3_^+^). The mass spectrum (Figure 4) demonstrated that the primary fragment ions corresponded to m/z of 412.3213, 138.1273, and 96.0798. The fragment ion at m/z 412.3213 (C_27_H_42_NO_2_, [M + H - H_2_O]^+^) resulted from the loss of H_2_O from the ion at m/z 430.3325. The fragment ion at m/z 138.1273 (C_9_H_6_N, [M + H - C_18_H_28_O_3_]^+^) was obtained by the loss of C_18_H_28_O_3_ from the precursor at m/z 430.3325; the ion detectable at m/z 96.0798 resulted from the loss of C_3_H_6_ from the ion at m/z 138.1273.

The retention time (t_R_) of the metabolite M1 was 2.179 min and was detected as a protonated molecular ion at m/z 446.3275 (C_27_H_43_NO_4_^+^), corresponding to a nominal increase in mass (16 Da) from that of sipeimine. The MS/MS spectrum (Figure 4) showed characteristic product ions at m/z 428.3165, 138.1273, and 96.0807. The fragment ion at m/z 428.3165 (C_27_H_42_NO_3_, [M + H - H_2_O]^+^) resulted from the loss of H_2_O from the ion at m/z 446.3275. Furthermore, the MS/MS spectrum was similar to that of the parent compound with respect to the ions at m/z 138.1273 and 96.0807. Therefore, M1 was identified as the hydroxylation product of sipeimine.

The metabolite M2 (t_R_ = 2.230 min) was detected as a protonated molecular ion at m/z 510.2899 (C_27_H_43_NO_6_S^+^). The difference in mass of 80 Da between sipeimine and M2 as well as the addition of the group SO_3_ indicated that M2 is the sulfate-conjugated metabolite of sipeimine. The MS/MS spectrum (Figure 4) revealed the characteristic fragment ions at m/z 412.3221 (corresponding to the loss of H_2_O and SO_3_ from the ion at m/z 510.2899) and 394.3108 (corresponding to the loss of H_2_O from the ion at m/z 412.3221). Furthermore, the MS/MS spectrum was similar to that of the parent compound with respect to the ion at m/z 138.1276. Therefore, M2 was identified as the sulfate-conjugated product of sipeimine.

The metabolite M3 (t_R_ = 2.138 min) corresponded to a molecular formula of C_33_H_53_NO_8_. The protonated molecular ion was observed at m/z 592.3878 [M + H]^+^, which is 162 Da higher than that of sipeimine. Additionally, the MS/MS spectrum (Figure 4) was identical to that of the parent drug with respect to the ions at m/z 412.3280 and 138.1308, indicating that M3 was the glucose conjugate of sipeimine. The MS/MS spectrum showed the characteristic fragment ions at m/z 574.3705 (corresponding to the loss of H_2_O from the ion at m/z 592.3878), 412.3280 (corresponding to the loss of C_6_H_10_O_5_ from the ion at m/z 574.3705), and 384.9542 (corresponding to the loss of CO from the ion at m/z 412.3221). Therefore, M3 was identified as the glucose-conjugated product of sipeimine.

### 3.6 Urinary and fecal excretion of sipeimine

**FIGURE 5:**
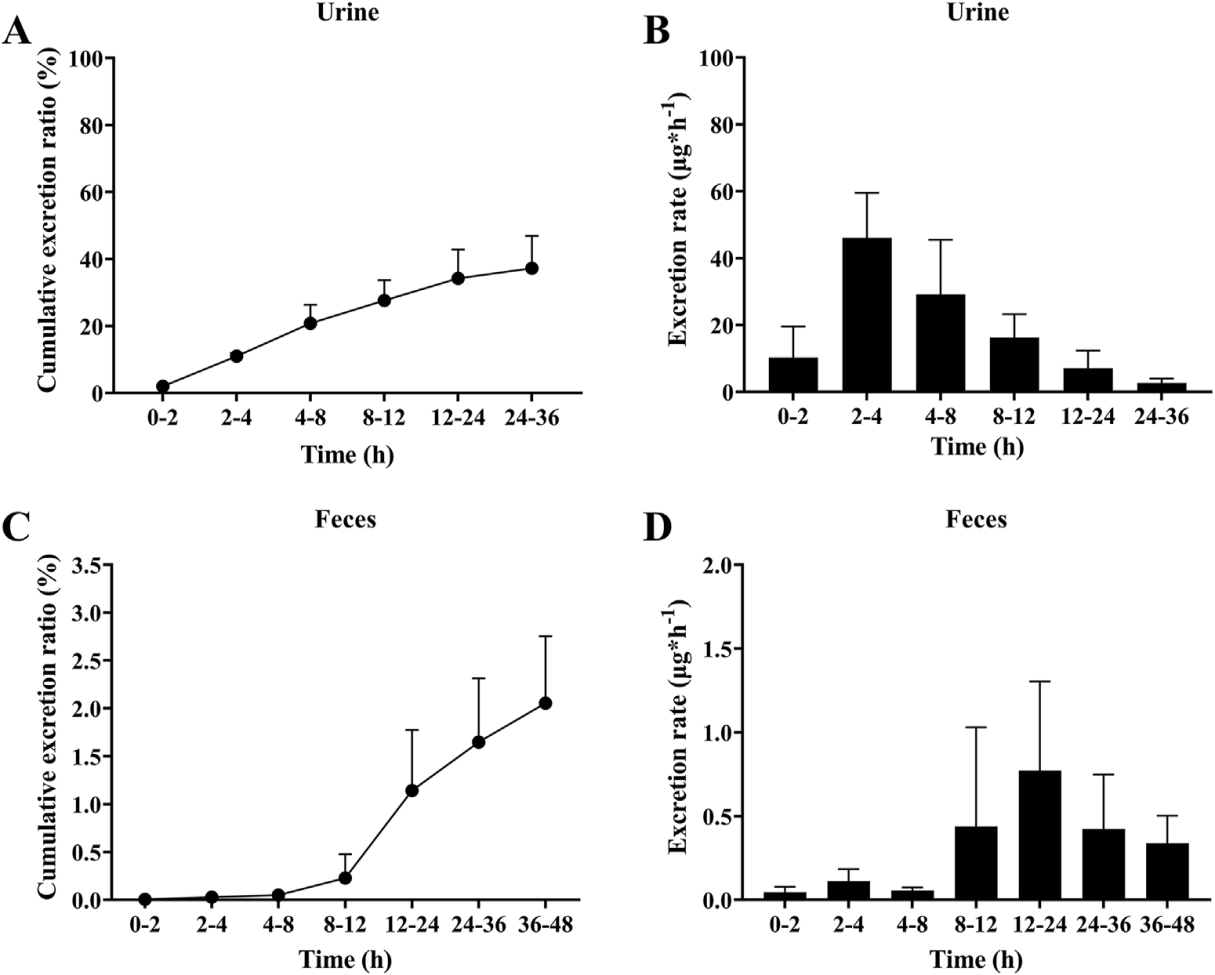
Excretion of sipeimine in urine **(A,B)** and feces **(C,D)** after intragastric administration of 5 mg/kg sipeimine (n = 6, Mean ± SD).

Drugs and their metabolites are excreted from the body through excretory processes, such as through renal, and intestinal excretions. The average cumulative excretion curves is shown in Figure 5A and in Figure 5C. After orally administering 5 mg/kg sipeimine to rats, the percentage of cumulative excretion of sipeimine in relation to the administered dose was found to be 37.28% ± 9.59% in urine at 36 h, and 2.65% ± 0.70% in feces at 48 h. The urinary excretion rate of sipeimine continued to increase throughout the detection period, with a peak urinary excretion rate of 46.01 ± 13.52 μg/h at the 2–4 h time point. The excretion rate in feces initially rises slowly and then followed by a rapid increase after 12 h, reaching a peak at 12–24 h at 0.77 ± 0.53 μg/h for feces. The excretion rate is displayed in Figure 5B and in Figure 5D. These findings indicated that sipeimine could be excreted through feces and urine, with urine excretion being the main route of elimination.

## 4 Conclusion

In conclusion, the study showcases the development of a rapid, reliable, and sensitive UPLC–MS/MS method and its application for the quantitation of sipeimine in biological samples collected from rats. The pharmacokinetics, tissue distribution, metabolism, and excretion of sipeimine in rats were systemically investigated. Sipeimine exhibited the characteristics of rapid absorption in the gastrointestinal tract, slow elimination, and high oral bioavailability. Following its absorption, sipeimine was rapidly distributed in all the organs except brain. The plasma protein binding ratio of sipeimine is moderate. The metabolism of sipeimine in rats is mainly accomplished via its hydroxylation, sulfation, and glucose conjugation. The excretion of sipeimine in its original form chiefly occurs via urine. These results are expected to prove useful for the interpretation of the pharmacokinetic and pharmacodynamic characteristics of sipeimine and the traditional Chinese medicines containing sipeimine.

## Data Availability

The original contributions presented in the study are included in the article/Supplementary Material, further inquiries can be directed to the corresponding authors.
