# Supplementary material for: Integrated UPLC–MS/MS and UPLC–Q–TOF–MS/MS analysis to reveal pharmacokinetics, tissue distribution, metabolism, and excretion of sipeimine in rats
Source: Front Pharmacol. 2025 May 30;16:1595731. doi: 10.3389/fphar.2025.1595731 (PMC12162304; doi:10.3389/fphar.2025.1595731)
Supplement: Supplementary file 1 [file Supplementaryfile1.docx]

**Supporting Information**

**Integrated UPLC–MS/MS and UPLC–Q–TOF–MS/MS analysis to reveal pharmacokinetics, tissue distribution, metabolism, and excretion of sipeimine in rats**

Author Names: Hui Zong ^a,1^, Chongyang Wang ^a,1^, Dongdong He ^a^, Liting Liu ^a^, Juanjuan Wan ^a^, Guodong Wang ^a^, Dingtao Li ^a^, Jun Ran ^a^, Meiling Zhang ^b, *^, Hui Tang ^a,**^, Liping Wang ^a,***^

**Contact Information:**

**Dr. Liping Wang** **(lead contact)**, Key Laboratory of Xinjiang Phytomedicine Resource and Utilization, Ministry of Education, School of Pharmacy, Shihezi University, Shihezi, Xinjiang, 832002, China. E-mail address: wangliping@shzu.edu.cn.

**Prof. Hui Tang**, Key Laboratory of Xinjiang Phytomedicine Resource and Utilization, Ministry of Education, School of Pharmacy, Shihezi University, Shihezi, Xinjiang, 832002, China. E-mail address: th_pha@shzu.edu.cn.

**Dr. Meiling Zhang**, Shihezi Institute for Drug Control, Shihezi, Xinjiang, 832000, China. E-mail address: 237554862@qq.com.

**Supplementary Methods**

**Bioanalytical Method Validation**

Method validation was based on the principles of the bioanalytical method validation guidelines, which including selectivity, linearity and lower limit of quantitation (LLOQ), precision and accuracy, stability, extraction recovery and matrix effect.

1. **Selectivity**

Selectivity was assessed by analyzing the chromatograms of blank biological samples, blank samples containing sipeimine standards, and rat biosamples after oral administration of sipeimine.

1. **Linearity and LLOQ**

Linearity was determined by analyzing the calibration curves. Calibration curves were constructed by plotting peak area ratios (sipeimine/IS) versus the concentration of the calibration solution. LLOQ was defined as the lowest calibration points in the calibration curve.

1. **Precision and Accuracy**

Three concentration levels of quality control samples in six replicates were analyzed within day (intra-day) and two consecutive days (inter-day) to assess the precision and accuracy. The precisions were defined as the relative standard deviation (RSD), and the accuracy was defined as the relative error (RE).

1. **Stability**

Three concentration levels of quality control (QC) samples in biological samples were used to evaluate the stability under different storage conditions, it included short-term stability (4 °C for 6 h), three freeze-thaw stability (from –20 °C to 25 °C), and long-term stability (–80 °C for 2 weeks).

1. **Extraction Recovery and Matrix Effect**

The extraction recovery and matrix effect of sipeimine were determined for three replicates at three different QC concentrations. The recovery was calculated by comparing the peak responses of the extracted QC samples with those of reference standards spiked with post-extracted blank matrix at the same concentration. The matrix effect was assessed by comparing the response of post-extracted blank matrix spiked with a known quantity of sipeimine that of standard solutions at equivalent concentrations.

**Supporting Figure Captions**

**Figure S1.** Representative MRM chromatograms of sipeimine in blank biological samples (A), blank biological samples spiked with sipeimine (B), and biological samples from rats after intragastrical administration of sipeimine (C). Biological samples include plasma, heart, liver, spleen, lung, and kidney.

**Figure S2.** Representative MRM chromatograms of sipeimine in blank biological samples (A), blank biological samples spiked with sipeimine (B), and biological samples from rats after intragastrical administration of sipeimine (C). Biological samples include brain, intestine, trachea, dialysate, urine, and feces.

**Figure S1**


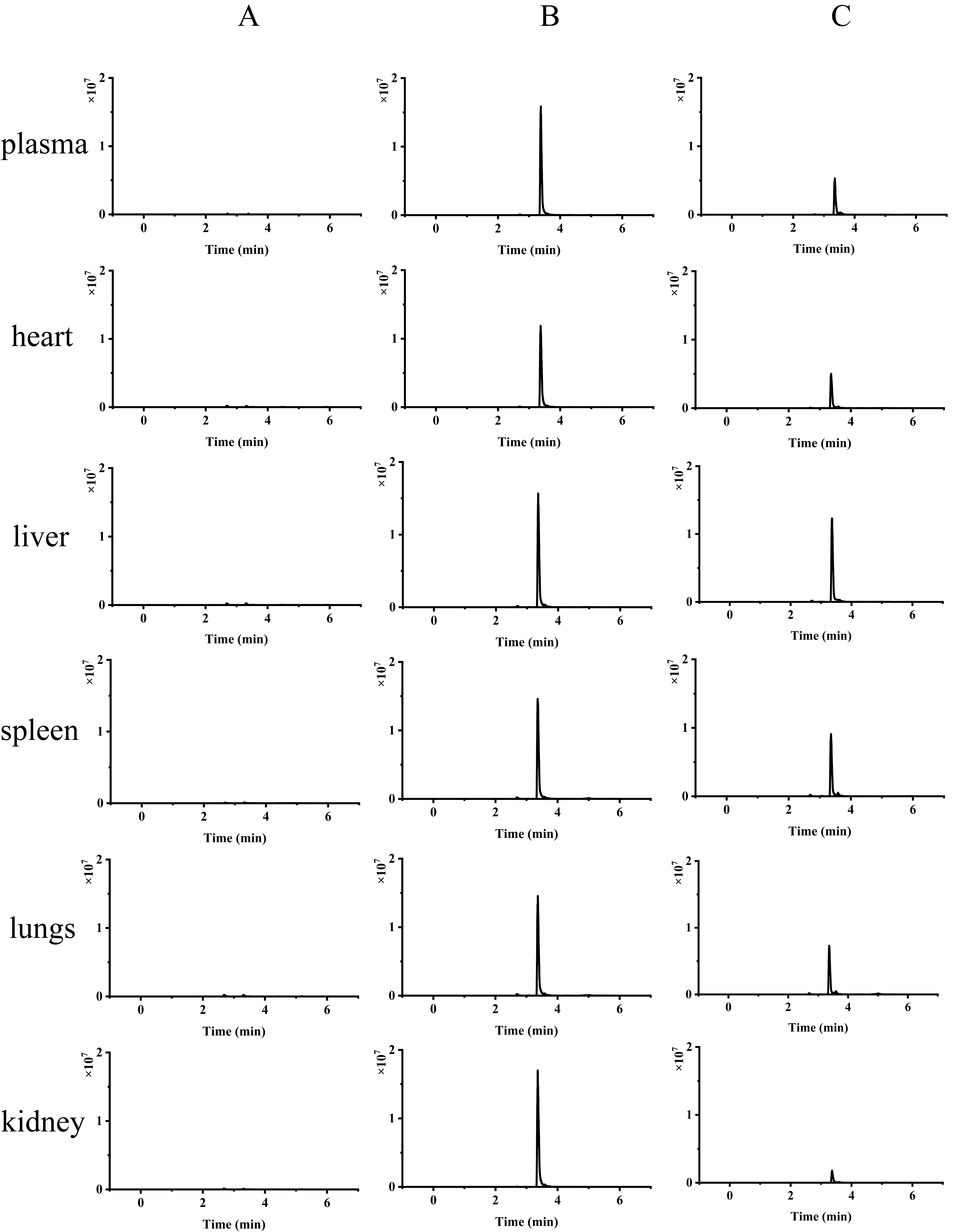


**Figure S2**

**
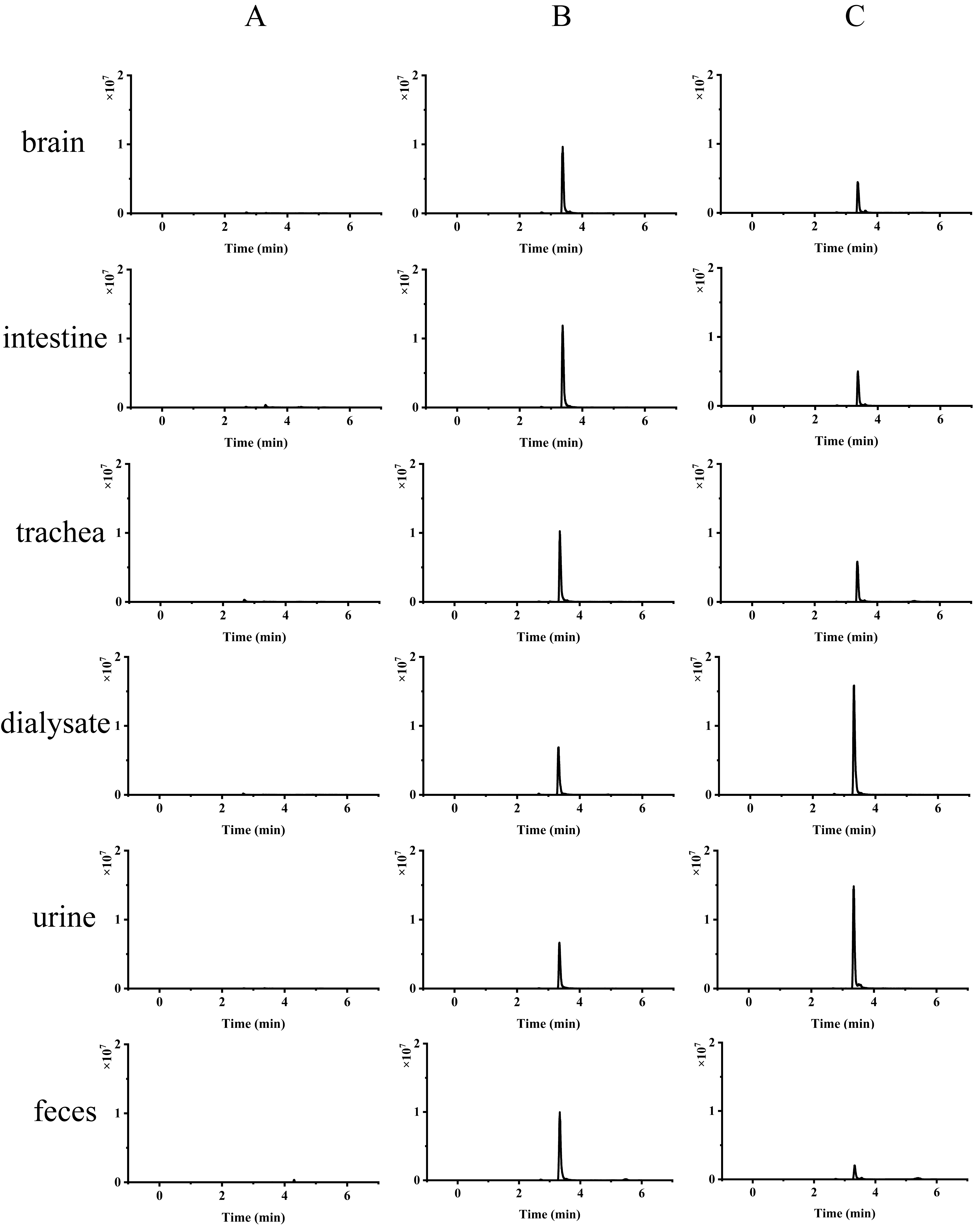
**

**Table S1. Calibration Curves,** **Correlation Coefficients, Linear Range, and LLOQs of Sipeimine in Rat Biological Samples.**

| matrix | calibration curves | r^2^ | linear range/nM | LLOQ/nM |
| --- | --- | --- | --- | --- |
| plasma | Y=0.0416274X+ 0.75092 | 0.9986 | 10-4000 | 10 |
| heart | Y=0.030136X+1.7596 | 0.9994 | 10-2000 | 10 |
| liver | Y=0.0325277X+1.76944 | 0.9999 | 10-4000 | 10 |
| spleen | Y=0.0351792X+1.47505 | 0.9997 | 10-4000 | 10 |
| lung | Y=0.0331479X+0.865935 | 0.9997 | 10-4000 | 10 |
| kidney | Y=0.0295675X+2.0807 | 0.9995 | 10-6000 | 10 |
| brain | Y=0.0457999X+0.768028 | 0.9984 | 10-400 | 10 |
| intestine | Y=0.0335674X+1.83685 | 0.9997 | 10-4000 | 10 |
| trachea | Y=0.029701X+7.77149 | 0.9998 | 10-6000 | 10 |
| dialysate | Y=0.0523974X+0.174902 | 0.9998 | 10-4000 | 10 |
| urine | Y=0.0345176X+0.0181607 | 0.9993 | 10-4000 | 10 |
| feces | Y=0.0467825X+7.60256 | 0.9995 | 10-4000 | 10 |

**Table S2. Precision and Accuracy of Sipeimine in Rat Biological Samples (n = 6).**

| matrix | concentration/nM | intra-day | | inter-day | |
| --- | --- | --- | --- | --- | --- |
|  |  | precision (RSD,%) | accuracy (RE,%) | precision (RSD,%) | accuracy (RE,%) |
| plasma | 25 | 5.6 | 12.2 | 12.2 | 11.6 |
|  | 250 | 11.9 | 10.1 | 9.3 | 12.1 |
|  | 2000 | 2.4 | 2.8 | 9.4 | 9.3 |
| heart | 50 | 5.6 | 6.6 | 6.3 | 5.7 |
|  | 250 | 1.1 | 0.9 | 2.1 | 1.5 |
|  | 1000 | 2.7 | 4.7 | 4.1 | 7.0 |
| liver | 50 | 12.2 | 9.9 | 16.7 | 14.3 |
|  | 250 | 2.9 | 4.7 | 1.6 | 10.9 |
|  | 2000 | 1.0 | 2.9 | 8.6 | 11.6 |
| spleen | 50 | 5.1 | 3.0 | 14.7 | 9.6 |
|  | 250 | 2.9 | 14.8 | 2.9 | 14.8 |
|  | 2000 | 0.8 | 0.7 | 8.9 | 6.9 |
| lung | 50 | 13.3 | 8.3 | 18.1 | 13.9 |
|  | 250 | 2.7 | 2.2 | 12.2 | 10.8 |
|  | 2000 | 3.5 | 5.3 | 9.1 | 11.8 |
| kidney | 100 | 10.4 | 10.1 | 8.6 | 8.3 |
|  | 1000 | 4.1 | 5.3 | 4.3 | 6.5 |
|  | 4000 | 2.2 | 2.4 | 1.9 | 1.6 |
| brain | 25 | 3.0 | 4.3 | 3.7 | 4.4 |
|  | 100 | 2.1 | 1.8 | 3.3 | 2.6 |
|  | 200 | 2.8 | 3.1 | 2.3 | 3.7 |
| intestine | 50 | 6.5 | 3.3 | 7.2 | 5.6 |
|  | 250 | 2.3 | 2.0 | 4.1 | 3.9 |
|  | 2000 | 2.2 | 2.3 | 5.5 | 5.6 |
| trachea | 100 | 10.8 | 10.4 | 12.3 | 9.2 |
|  | 1000 | 1.7 | 3.2 | 2.6 | 4.6 |
|  | 4000 | 1.4 | 1.2 | 2.5 | 2.0 |
| dialysate | 25 | 3.7 | 3.5 | 3.3 | 2.8 |
|  | 250 | 1.9 | 1.6 | 2.0 | 1.7 |
|  | 2000 | 2.2 | 2.4 | 2.7 | 2.5 |
| urine | 50 | 3.9 | 3.5 | 9.0 | 8.1 |
|  | 250 | 4.2 | 3.7 | 4.0 | 3.4 |
|  | 2000 | 4.5 | 5.3 | 4.0 | 6.8 |
| feces | 25 | 12.6 | 11.5 | 12.6 | 12.1 |
|  | 250 | 3.2 | 3.1 | 4.7 | 5.2 |
|  | 2000 | 3.8 | 3.2 | 5.0 | 4.6 |

**Table S3. Stability of Sipeimine in Rat Biological Samples (n = 3).**

| matrix | concentration/nM | freeze-thaw cycles | | short term | | long term | |
| --- | --- | --- | --- | --- | --- | --- | --- |
|  |  | RSD  (%) | RE  (%) | RSD  (%) | RE  (%) | RSD  (%) | RE  (%) |
| plasma | 25 | 7.0 | 4.9 | 6.9 | 4.9 | 4.8 | 4.3 |
|  | 250 | 1.9 | 1.6 | 0.3 | 1.0 | 1.5 | 1.6 |
|  | 2000 | 6.7 | 5.0 | 3.9 | 2.9 | 2.2 | 1.8 |
| heart | 50 | 5.6 | 5.9 | 17.5 | 13.2 | 3.9 | 3.8 |
|  | 250 | 2.2 | 1.7 | 3.6 | 3.1 | 2.6 | 2.4 |
|  | 1000 | 2.1 | 1.7 | 1.6 | 1.0 | 1.1 | 0.9 |
| liver | 50 | 5.1 | 8.8 | 8.3 | 8.3 | 1.3 | 5.0 |
|  | 250 | 1.5 | 1.6 | 4.2 | 2.8 | 3.9 | 4.4 |
|  | 2000 | 4.8 | 4.5 | 3.9 | 3.7 | 2.0 | 4.4 |
| spleen | 50 | 9.5 | 9.6 | 2.8 | 9.1 | 6.4 | 9.9 |
|  | 250 | 1.0 | 0.8 | 2.3 | 1.6 | 1.8 | 1.5 |
|  | 2000 | 2.2 | 1.9 | 2.3 | 1.5 | 0.3 | 1.3 |
| lung | 50 | 4.0 | 3.8 | 4.1 | 3.2 | 3.9 | 3.7 |
|  | 250 | 1.2 | 2.1 | 2.4 | 1.5 | 1.8 | 1.6 |
|  | 2000 | 5.1 | 3.5 | 2.1 | 2.0 | 4.8 | 3.5 |
| kidney | 100 | 1.4 | 1.4 | 3.4 | 2.6 | 2.6 | 1.8 |
|  | 1000 | 1.1 | 5.2 | 2.5 | 5.1 | 1.0 | 5.7 |
|  | 4000 | 0.7 | 1.6 | 0.7 | 0.6 | 3.2 | 2.1 |
| brain | 25 | 4.0 | 4.1 | 7.9 | 6.1 | 9.0 | 9.0 |
|  | 100 | 0.2 | 2.1 | 6.1 | 5.4 | 5.1 | 4.7 |
|  | 200 | 7.7 | 6.0 | 4.6 | 3.7 | 0.3 | 3.6 |
| intestine | 50 | 5.5 | 6.4 | 4.6 | 3.9 | 6.9 | 11.8 |
|  | 250 | 3.5 | 2.6 | 2.3 | 1.9 | 1.9 | 1.6 |
|  | 2000 | 5.7 | 5.9 | 4.2 | 5.7 | 4.7 | 5.0 |
| trachea | 100 | 4.8 | 3.3 | 8.5 | 8.8 | 15.1 | 12.7 |
|  | 1000 | 5.1 | 3.5 | 0.7 | 6.4 | 3.6 | 4.9 |
|  | 4000 | 1.5 | 2.0 | 2.0 | 1.7 | 1.0 | 1.5 |
| dialysate | 25 | 2.5 | 2.1 | 2.3 | 3.8 | 4.9 | 4.6 |
|  | 250 | 0.2 | 1.9 | 1.1 | 1.1 | 1.8 | 1.4 |
|  | 2000 | 1.7 | 4.2 | 1.7 | 3.3 | 4.0 | 3.2 |
| urine | 50 | 11.2 | 9.2 | 7.1 | 9.8 | 4.2 | 2.7 |
|  | 250 | 3.3 | 2.3 | 4.6 | 3.7 | 4.7 | 3.7 |
|  | 2000 | 3.5 | 2.4 | 2.8 | 5.8 | 1.6 | 2.6 |
| feces | 25 | 9.2 | 6.4 | 18.0 | 12.8 | 13.6 | 13.6 |
|  | 250 | 0.3 | 3.4 | 5.8 | 5.3 | 0.7 | 5.1 |
|  | 2000 | 1.8 | 5.3 | 2.7 | 7.4 | 4.3 | 4.7 |

**Table S4. Extraction Recovery and Matrix Effect of Sipeimine in Rat Biological Samples (n = 3).**

| matrix | concentration/nM | extraction recovery  mean ± SD (%) | matrix effect  mean ± SD (%) |
| --- | --- | --- | --- |
| plasma | 25 | 80.0 ± 5.4 | 79.0 ± 0.9 |
|  | 250 | 80.0 ± 5.4 | 90.0 ± 0.4 |
|  | 2000 | 85.1 ± 7.0 | 80.4 ± 1.2 |
| heart | 50 | 75.8 ± 8.8 | 78.0 ± 0.3 |
|  | 250 | 79.8 ± 3.7 | 82.7 ± 1.5 |
|  | 1000 | 87.0 ± 2.2 | 87.3 ± 0.5 |
| liver | 50 | 85.7 ± 6.3 | 89.0 ± 8.7 |
|  | 250 | 92.6 ± 3.9 | 90.6 ± 2.1 |
|  | 2000 | 98.1 ± 1.9 | 93.3 ± 4.0 |
| spleen | 50 | 98.7 ± 9.3 | 82.6 ± 6.8 |
|  | 250 | 89.4 ± 5.2 | 88.6 ± 2.8 |
|  | 2000 | 95.3 ± 2.3 | 90.9 ± 1.6 |
| lung | 50 | 85.4 ± 3.9 | 92.5 ± 2.5 |
|  | 250 | 90.3 ± 5.3 | 94.9 ± 2.7 |
|  | 2000 | 93.3 ± 2.4 | 89.2 ± 3.1 |
| kidney | 100 | 88.7 ± 3.2 | 90.2 ± 2.6 |
|  | 1000 | 90.5 ± 5.5 | 89.0 ± 0.8 |
|  | 4000 | 93.5 ± 0.6 | 89.3 ± 1.4 |
| brain | 25 | 76.3 ± 7.3 | 102.3 ± 7.2 |
|  | 100 | 91.4 ± 4.0 | 86.3 ± 5.6 |
|  | 200 | 91.7 ± 3.5 | 90.6 ± 1.4 |
| Intestine | 50 | 86.0 ± 5.5 | 95.2 ± 0.5 |
|  | 250 | 92.9 ± 1.8 | 90.9 ± 2.3 |
|  | 2000 | 92.2 ± 3.7 | 90.2 ± 4.2 |
| trachea | 100 | 76.9 ± 1.7 | 96.7 ± 3.1 |
|  | 1000 | 77.4 ± 3.7 | 84.9 ± 2.0 |
|  | 4000 | 84.7 ± 0.9 | 89.1 ± 1.4 |
| dialysate | 25 | 89.5 ± 5.3 | 90.1 ± 4.3 |
|  | 250 | 95.0 ± 1.3 | 93.5 ± 1.7 |
|  | 2000 | 93.2 ± 1.2 | 96.0 ± 2.0 |
| urine | 50 | 85.2 ± 6.4 | 91.9 ± 9.4 |
|  | 250 | 92.4 ± 2.7 | 94.2 ± 2.7 |
|  | 2000 | 94.0 ± 7.9 | 96.6 ± 1.7 |
| feces | 25 | 106.1 ± 9.3 | 96.0 ± 9.3 |
|  | 250 | 108.3 ± 5.6 | 97.7 ± 3.6 |
|  | 2000 | 101.6 ± 1.4 | 83.2 ± 3.4 |
